# SRL-Coated PAMAM Dendrimer Nano-Carrier for Targeted Gene Delivery to the Glioma Cells and Competitive Inhibition by Lactoferrin 

**Published:** 2016

**Authors:** Amir zarebkohan, Farhood Najafi, Hamid Reza Moghimi, Mohammad Hemmati, Mohammad Reza Deevband, Bahram Kazemi

**Affiliations:** a Biomedical Engineering and Medical Physics Department, Faculty of Medicine, Shahid Beheshti University of Medical Sciences, Tehran, Iran.; b Department of Resin and Additives, Institute for Color Science and Technology, Tehran, Iran.; c School of Pharmacy, Shahid Beheshti University of Medical Sciences, Tehran, Iran.; d Cellular and Molecular Biology Research Center, Shahid Beheshti University of Medical Sciences, Tehran, Iran.; e Department of Biotechnology, Faculty of Medicine, Shahid Beheshti University of Medical Sciences, Tehran, Iran.

**Keywords:** SRL peptide, PAMAM dendrimer, Gene delivery, C6 glioma, Targeted delivery, Brain tumor

## Abstract

Glioma, as a primary tumor of central nervous system, is the main cause of death in patients with brain cancer. Therefore, development of an efficient strategy for treatment of glioma is worthy. The aim of the current study was to develop a SRL peptide-coated dendrimer as a novel dual gene delivery system for targeting the LRP receptor, an up-regulated gene in both BBB and glioma cells.

To perform this investigation, our newly developed nanocarrier (PAMAM-PEG-SRL) was used for gene delivery to C6 glioma cell lines. DNA (GFP) was loaded in these functionalized nanoparticles and their cellular uptake/distribution and gene transfection efficacy was evaluated by fluorescence and confocal microscopy. In vitro studies showed that SRL-modified nanoparticles have good transfection efficacy. Results revealed improved gene transfection efficiency of newly-synthesized delivery system. We also found that lactoferrin, as a LRP ligand, reduced the gene transfection efficacy of the delivery system due to its higher affinity compared to SRL peptides (Competitive inhibition). The present results suggest that the synthesized delivery system has the potential to be used as an alternative targeted drug delivery system for brain tumors.

## Introduction

Glioma, as a primary tumor of the central nervous system, is responsible for most of brain cancer deaths (1). The ability of glioma cells to invade surrounding normal tissues makes complete surgical resection impossible, in turn leading to tumor recurrence (2). In view of this, there is an urgent need to develop an effective therapeutic delivery system to selectively deliver cytotoxic drugs to tumor cells while sparing normal cells. However, poor blood–brain barrier (BBB) penetration of drugs is a major obstacle for targeted delivery of drugs and genetic materials, such as DNA, to brain tumors. This has made the treatment of nervous system diseases rather difficult and complicated. The blood–brain barrier prevents the entry of most drugs and genetic materials (almost 98%) into the brain. Generally, only small (less than 400 Da) or high-lipophilic particles are able to pass through the blood–brain barrier. In fact, poor blood–brain barrier penetration are considered to be the most common causes of treatment failure in patients with nervous system diseases (3, 4). Moreover, it seems to be important to prevent the intracellular accumulation of therapeutic agents in normal tissues and to increase the concentration of these agents at tumor sites (5). 

Nowadays, nanotechnology provides solutions to such problems, so that a wide variety of nanoparticles and supramolecular nanodevices have been developed to improve the penetration of drugs and even genetic materials across different endothelial and epithelial barriers in a non-invasive manner (6, 7). PAMAM dendrimers, a class of the nanoparticles, have been considered as a promising system for gene delivery (8, 9). More importantly, due to the presence of primary surface amines in this type of nanoparticles, PAMAM dendrimers have an intrinsic capacity to bind to a variety of negatively-charged agents, including gene segments, plasmids and several types of ligands (8). However, these carriers cannot easily permeate the BBB. One possible way that has been used to circumvent this problem is the specific binding of certain ligands to the surface of these nanoparticles to facilitate selective passage of nanoparticles across the BBB.

A dual-targeting strategy is a newly-introduced method recently developed for the targeted delivery of therapeutic agents to the brain, in which nanocarriers decorated with only one targeted ligand on their surface are applied to target the glioma cells. In this approach, nanocarriers are able to deliver therapeutic and imaging agents to tumors by targeting specific receptors overexpressed on the surface of the BBB and glioma cells (10, 11). A variety of ligand types has been used for this purpose, including lactoferrin (11), Angiopep (9), Chlorotoxin (12), RGD (13), LDLR-mediated peptide-22 (14) and T7 peptide (15). Among them, lactoferrin and Angiopep target the LRP present on the surface of brain capillary endothelial cells (BCECs) (9, 11). Lipoprotein endocytosis is mediated by three structurally related cell surface receptors, which the presence of at least one copy of an NPXY motif in their structure leads to the receptor internalization into coated pits (16). Prototype of this family is low density lipoprotein (LDL)’ receptor (17). These receptors bind to apoB-100 or apoE in plasma lipoproteins and remove them from plasma.

LRP, a member of the LDL receptor gene family, is much larger than the LDL receptor (16). The physiological role of LRP receptors include the permeability control of the BBB, regulation of vascular tone and postischemic lesion formation, in response to activated tissue-type plasminogen activator (18). LRP is expressed in all cells, and binds to more than 40 different types of ligands such as lipoproteins, protease/protease-inhibitor complexes, extracellular matrix proteins, viruses, growth factors and cytokines (19). Also, its demonstrated that lactoferrin is a specific ligand of LRP (16, 20). 

The unique feature of this protein is a high transcytosis rate (21). 

It is well-documented that LRP is overexpressed on glioma cells (10, 22, 23). Therefore, LRP can be served as a potential dual targeting receptor to overcome the BBB and delivery of therapeutic agents to glioma cells.

More recently, we have developed a new nanocarrier based on the G4 PAMAM dendrimer, synthesized by binding the SRL peptide (CLSSRLDA) to G4 PAMAM dendrimers (24). The SRL peptide, designed by Pasqualini and Ruoslahti, exhibited high penetration efficiency into brain capillary endothelial cells (BCECs) (25). In our previous study, we showed that the SRL peptide was found to be a LRP ligand, and nanocarriers modified by this ligand were able to internalize into the BCECs by the clathrin/caveolin energy-dependent endocytosis mechanism (24). 

Because of the fact that C6 glioma cells overexpressed LRP, we sought to attach the SRL peptide (with the CLSSRLDA sequence) on the surface of G4 PAMAM dendrimers, as a dual targeting delivery system, for targeting LRPs present on the C6 glioma cell line. Also, in this study the inhibitory effect of lactoferrin on the entrance of PAMAM-PEG-SRL/DNA complexes to C6 glioma cells was evaluated.

## Materials and Methods

The pEGFP-N1 plasmid (Clontech, Palo Alto, CA, USA) was purified using a QIAGEN Plasmid Mega Kit (Qiagen GmbH, Hildden, Germany). The SRL peptides (99% purity), both CLSSRLDA and LSSRLDAC sequences, were purchased from Biomatic Company (Wilmington, USA). The PAMAM G4 dendrimer was synthesized by an in-situ branch cell method, a two-step iterative process to construct poly (amidoamine) (PAMAM) dendrimers possessing either terminal ester or amine groups. The basis of the method involves (a) alkylation with methyl acrylate, and (b) amidation with ethylenediamine (26). Lactoferrin, filipin complex (from Streptomyces filipinesis), L-polylysin (30000-70000MW) and phenylarsine oxide were bought from Sigma-Aldrich (St. Louis, MO, USA). Bifunctional PEG (NHS-PEG-MAL, MW 1000) was obtained from Nanocsinc (Boston, USA). BODIPY fluorophore (4,4-difluoro-5,7- dimethyl-4-bora-3a,4a-diaza-s-indacene-3-propionic acid, sulfosuccinimidylester,sodium salt) was purchased from Molecular Probes (Eugene, OR, USA).

*Cell line*

Rat Brain capillary endothelial cells (BCECs) were kindly provided from Dr. Aghaei (Neuroscience Research Center, Shahid Beheshti University of Medical Sciences), and cultured according to ATCC guidelines. BCECs were cultured in a Rat Brain Endothelial Cell Growth Medium (Cell Applications Inc, San Diego CA, USA), supplemented with 20% fetal calf serum (FCS), epidermal growth factor (100 mg/mL), L-glutamine (2 mmol/L), heparin (20 mg/mL), insulin (40 mU/mL), penicillin (100 U/mL) and streptomycin (100mg/mL) under standard conditions in a humidified 5% CO2 incubator at 37 ^o^C.

The C6 glioma cell line was purchased from National Cell Bank of Iran (NCBI), Pasteur Institute, Tehran, Iran. C6 glioma cells were cultured in DMEM (Sigma–Aldrich), supplemented with 10% FBS, 100 U/mL penicillin and 100 g/mL streptomycin at 37 ^o^C in a humidified incubator containing 5% CO2. All the cells used in this study were extended between 10 to 15 passages.

*Synthesis of PAMAM derivatives, and Characterization of Nanoparticles*

PAMAM derivatives were synthesized according to the same methods described in the previous papers (27, 28). In brief, PAMAM-PEG-SRL at 1:6:3 ratio synthesized as previously described. And their size and physicochemical characteristics was evaluated by NMR, Zeta sizer and UV spectroscopy (24).

*Preparation of Dendrimer/DNA Nanoparticles*

PAMAM and PAMAM-PEG-SRL were freshly synthesized, and diluted in distilled water. Then, a DNA solution (100 mg DNA/mL in 50 mM Sodium sulfate solution) was added to nanoparticle solution at specific weight ratio (1:10, PAMAM/DNA w/w), and gently vortexed at 37^ o^C for 30 sec. Afterwards, the complex was incubated at room temperature for 30 min to allow self-assembly formation. Agarose gel electrophoresis was carried out to verify the formation and stability of the nanoparticle-DNA complex (24).

*Preparation of endocytosis inhibitors*

Filipin and phenylarsine oxide were dissolved in dimethyl sulfoxide (DMSO), and then diluted in PBS (pH 7.4) to obtain 0.5 mg/mL. In addition, SRL (100 mg/mL), colchicines (2.5 mM) and lactoferrin (1 mg/mL) were prepared in water and then diluted in PBS, pH 7.4 (27).

*Cellular uptake of dendrimers*

BCECs and C6 glioma cells were seeded at a density of 2×10^4^ cells/well in 24-well plates (Corning- Coaster, Sigma, USA), for 48 hours. Cell confluence and morphology were checked under a fluorescent microscope throughout the experiment. Afterwards, the C6 glioma cells were incubated at a 1 µM concentration of BODIPY-labeled PAMAM or 0.2, 0.5 and 1.0 µM concentrations of BODIPY-labeled PAMAM–PEG–SRL at 37^ o^C for 30 minutes with a final concentration of 1 µM. Furthermore, to study the energy dependency of nanoparticles internalization into target cells, C6 glioma cells were exposed to BODIPY-labeled PAMAM–PEG–SRL at a density of 1µM at 4 ^o^C. The culture supernatant was removed, and cells were then washed three times with cold PBS (pH 7.4), and examined using a fluorescent microscope (Olympus, Osaka, Japan). To quantitatively analyze the cellular uptake of PAMAM-PEG-SRL at different concentrations, the cells BCECs and C6 glioma were cultured at a density of 8×10^4^ cells/well in 6-well plates (Corning-Coaster, Sigma, USA) for 72 h. After checking the cell confluence and morphology, the cells were incubated at a 1 µM concentration of BODIPY-labeled PAMAM–PEG–SRL for 30 minutes. As noted in the previous section, the cells were washed three times with PBS (pH 7.4), and then, the treated cells were trypsinized and centrifuged for 8 minutes at 1600 rpm to obtain the cellular pellet. The pellet was resuspended in PBS (pH 7.4), and analyzed by a flow cytometer (FACS Calibur, BD Biosciences, Bedford MA, USA) equipped with an Argon Ion Laser (488 nm) as a stimulating source of fluorescent. For each sample, 10000 events were collected and analyzed using WinMDI software. Cells cultured under normal conditions and live cells were considered as controls and gates, respectively. In general, the only cells at the gate were analyzed. The mean density of fluorescent cells was calculated as a histogram plot (27).

*Gene transfer efficiency into C6 glioma cells*

The Plasmid DNA was covalently labeled using the fluorescent dye ethidiummonoazide bromide (EMA). Briefly, the Plasmid DNA solution (1 mg/mL in TE buffer, pH 7.0) was diluted in EMA solution until reaching a concentration of 0.1 mg/mL, and then incubated for 30 min in darkness. Then, the solution was precipitated by the addition of ethanol to a final concentration of 30% (v/v). The pellet was collected by centrifugation at 12000 rpm for 20 min, and resuspended in 50 mM sodium sulfate. EMA-DNA solution was used to prepare nanoparticles by the same method. C6 glioma cells were seeded at a density of 2.0 ×10^4^ cells/well in 24-well plates, and then incubated for 48 hours. Subsequently, the nanoparticles (PAMAM or PAMAM-PEG-SRL) containing EMA-DNA were added to the cells (27). Thirty minutes later, the culture supenatant was removed, and the remaining cells were washed 3 times with PBS (pH 7.4), and observed under a fluorescent microscope.

*Cellular uptake mechanism of dendrimer/DNA nanoparticles*

C6 glioma cells were seeded at a density of 2×10^4^ cells/well in 24-well plates, and cultured using the above method. After monitoring the morphology and confluency of the complex filipin (0.5 mg/mL), phenylarsine oxide (2.5 mM), colchicines (2.5 mM) or lactoferrin (1mg/ml) were added to each well, and incubated for 10 minutes (27). Subsequently, the supernatants were removed, and an equal volume of the nanoparticle PAMAM-PEG-SRL containing EMA-DNA were added to the above mixtures. After 30 minutes, the supernatant was removed, and the cells were washed three times with cold PBS (pH 7.4), and observed under a fluorescent microscope. 

*Confocal microscopy*

The intracellular distribution of the nanoparticles was studied using a confocal microscopy. C6 glioma cells were seeded at a density of 10^4^ cells/well in a 35 mm glass-bottom culture dish, and cultured at 37 ^o^C under an atmosphere containing 5% CO2 for 48 hours, recultured in serum-free media, and then assessed 15 and 60 min after exposed to the PAMAM-PEG-SRL/DNA complex (5 µg DNA/well, N/P=10). After this time, the cells were washed with cold PBS, and subjected to 50nM LysoTracker Green (Molecular probes Inc.) and DAPI for 30 and 10 minutes, respectively. Afterwards, the cells were washed three times with cold PBS, fixed with paraformaldehyde and observed under confocal laser scanning microscopy (29).

*Qualitative assessment of Green Fluorescence protein expression (GFP) in transfected cells.*

 C6 glioma cells were cultured at a density of 1.0 × 10^6^ cells/well in 6-well plates in 2 mL of culture media for 18 h to reach 50% confluence for gene therapy experiments. Prior to transfection, the medium was replaced with a serum-free medium, and the cells were then incubated for 4 h with the complexes PAMAM-PEG-SRL/DNA and PAMAM/DNA in the presence or absence of lactoferrin (8 micrograms per well, 8 μg/well) (Figure 8). The medium was replaced with a fresh medium containing 10% FBS, and then incubated again for 24 hours. GFP expression levels were detected under a fluorescent microscope (Olympus, Osaka, Japan) (30).

*Luciferase Activity Assay.*

C6 glioma cells were cultured in a 24/well plate at the appropriate confluence for 24 h, and allowed to grow 50% confluence at the transfection time. Prior to transfection, the medium was replaced with serum-free medium, and the cells were then incubated with PAMAM-PEG-SRL/pDNA complexes (4 μg /well) at a specific weight ratio under standard conditions for 4 h.  Subsequently, the medium was replaced with fresh a medium containing 10% serum, and incubated again for 24 h.

To measure the luciferase activity, cells were washed with PBS two times, and 100 to 200 µL of cell lysis buffer was added to each well for 5 minutes at 37 °C. Cell lysis solution was centrifuged at 1200 rpm for 5 min, 10 µL of the supernatant was mixed with 25 µL of both luciferase substrate and ATP solution, and luciferase activity was measured with Sirius luminometer (Autolumat LB953, EG and G, Berthold, Germany). Small amounts of the protein were determined by the bicinchoninic acid assay (Bio-Rad Laboratories, Hercules, CA). Relative light units (RLUs) were normalized with the concentration of the proteins extracted from the cells. All transfection experiments were performed as triplicate. The transfection activity was reported by RLUs (31). Lipofectamine was used as a control to compare each sample.

*Statistical analysis*

All quantitative assessments were carried out in quadruplicate, and the experiments were repeated 3 times. The data were expressed as mean ± SD. Statistical analysis was performed by the one-way ANOVA, using the statistical software.

## Results

*In-vitro uptake of DNA-containing particles by C6 glioma cells*

**Figure 1:**
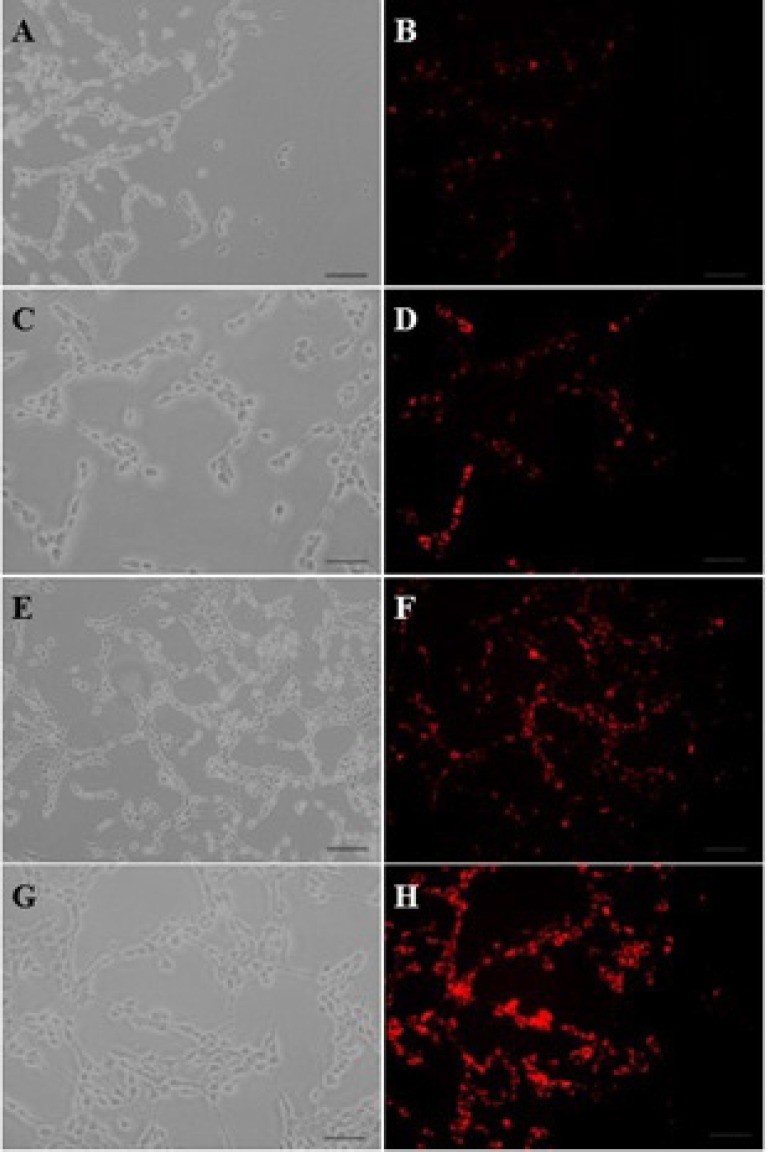
The fluorescent images of PAMAM–PEG–SRL/ EMA-labeled DNA cellular internalization. Concentration of PAMAM of samples was increased from 50 μg/ml (A, B) to 250 μg/ml (G, H). Bar: 50μm

**Figure 2:**
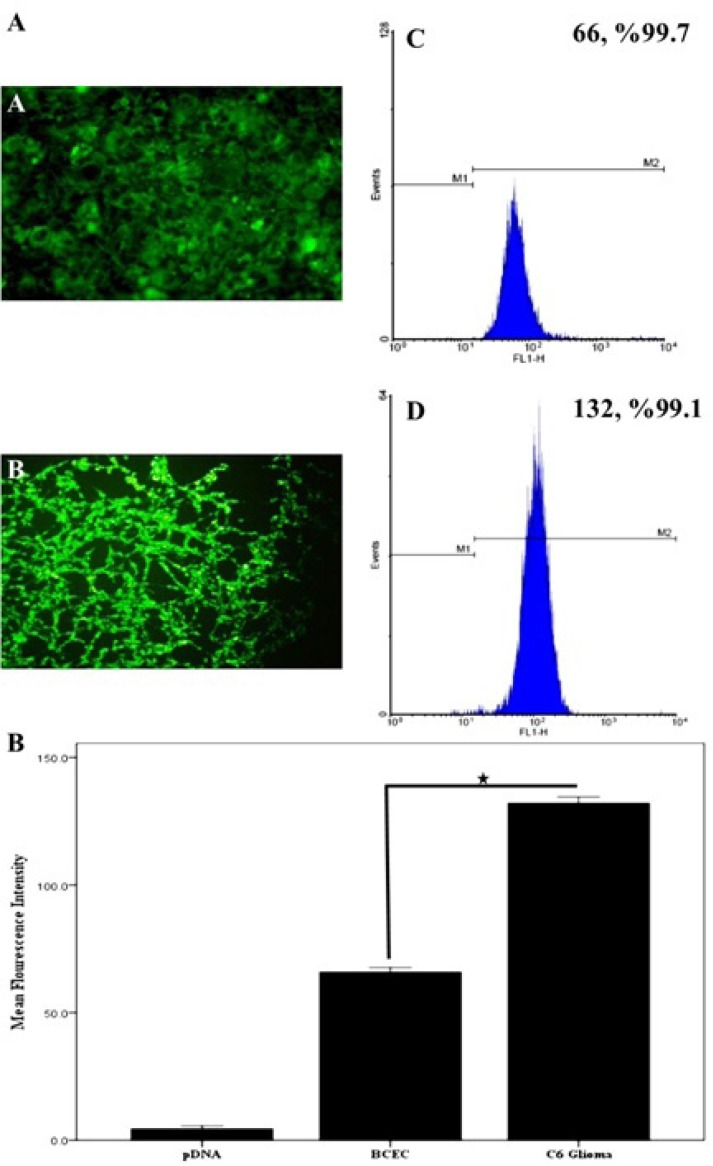
A) Cellular internalization of BODIPY-labeled PAMAM–PEG–SRL in BCEC and C6glioma cells. The concentration of PAMAM adjusted to 1.0 μM (250 μg/ ml). Cellular uptake of BODIPY-labeled PAMAM-PEG-SRL nanoparticles was examined after 30 min incubation by fluorescent. Flow cytometry analysis of BCECs and C6 glioma cells after a 30min incubation. Histogram of normal BCECs (a), and C6 glioma cells (b). C) After setting a gate according to the control the number of BODIPY-positive cells were analyzed. Mean fluorescence intensity was shown. *P value <0.05

The EMA-labeled nanoparticles were used for the study of cellular uptake, and the fluorescent images were analyzed by a fluorescent microscope. To investigate the concentration dependence of nanoparticles internalization into C6 glioma cells, the cells were measured after a 30-min cell exposure to different concentrations (0.2 to 1 µM) of EMA-labeled PAMAM-PEG-SRL/DNA. As it can be seen in Figure 1, an increase in the complex concentration up to 1 µM (250 µg/mL) resulted in an outstandingly higher cellular uptake of PAMAM-PEG-SRL/DNA (Figure 1G, H), demonstrating the dose-dependency entrance behavior of the complexes. At the 1 µM (250 µg/ml) concentration of PAMAM-PEG-SRL/DNA, there was an equal amount the positive-BODIPY cells (data not shown), while mean fluorescent intensity increased from 66 for BCEC to 132 for C6 glioma cells (Figure 2B). An increase in mean fluorescent intensity is due to the higher expression level of LRP in C6 glioma cells than BCEC, therefore which can easily be targeted by the SRL peptide (as a LRP ligand) present on the surface of nanoparticles. 

**Figure 3:**
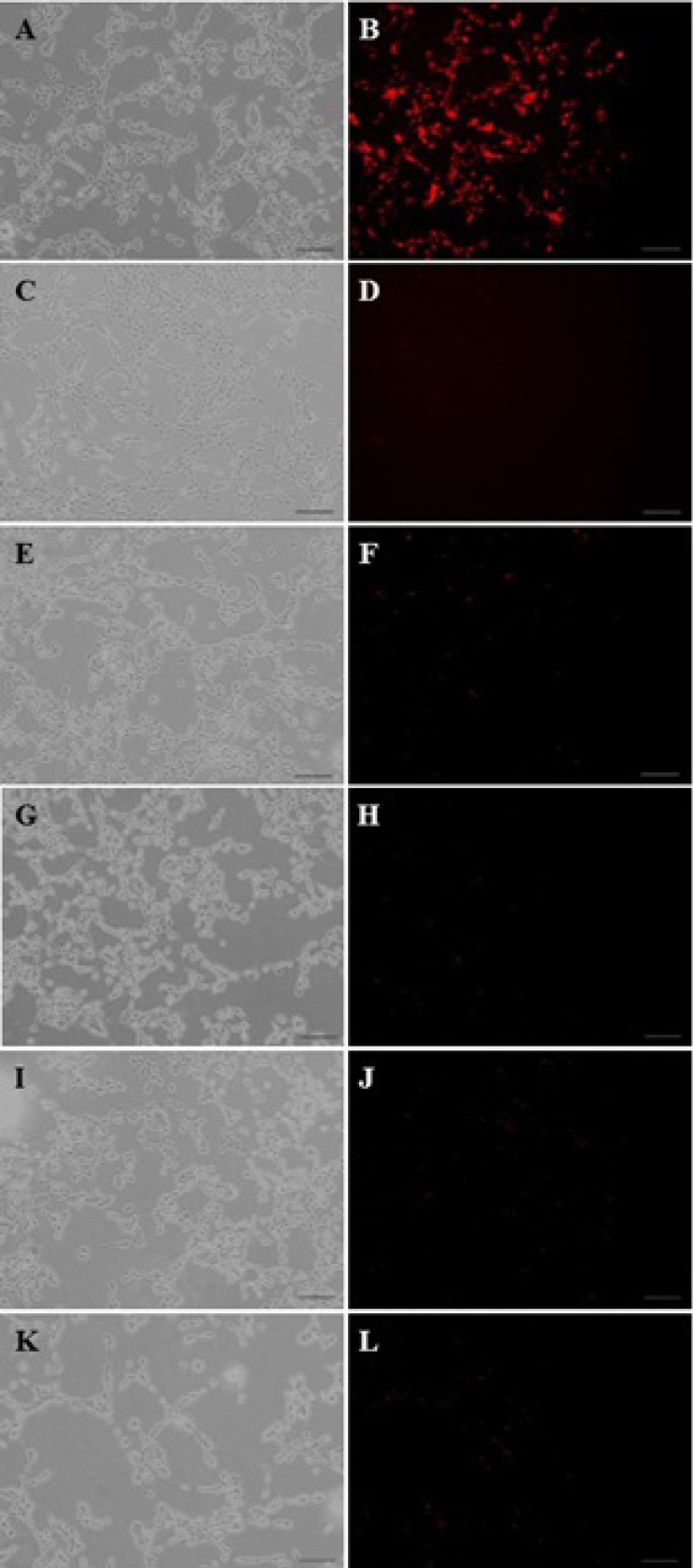
The fluorescent images of PAMAM–PEG–SRL/ EMA-labeled DNA cellular internalization. Internalization mechanism of nanoparticles by C6 glioma cells. Concentration of PAMAM of all samples was adjusted to 1μM (250 μg/m). PAMAM–PEG–SRL/EMA-labeled DNA without any inhibitor (as control) (A, B). Cells were incubated with different endocytosis inhibitors including: lactoferrin (E, F), phenylarsine oxide (G, H), filipin complex (I, J), colchicine (K, L), and at 4^o ^C (C, D); for 30 min. Red: EMA-labeled DNA. Bar: 50μm

The cellular uptake of EMA-labeled PAMAM-PEG-SRL/DNA at 4 ^o^C was much less than 37 ^o^C that could be considered as another reason for energy-dependency of nanoparticles cellular uptake, endocytosis (Figure 3C, D) (32). Results also indicated that the cellular uptake of the modified nanoparticles is dose-dependent.

*The exact cellular uptake mechanism of DNA-containing particles*

Similar to our previous experiment (24), the results from this study showed that a ratio of 1:6:3 showed the highest level of uptake (data not shown) amongst the three PAMAM:PEG:SRL compositions used here (1:2:1, 1:4:2 and 1:6:3 molar ratios). Furthermore, it was shown that the cellular uptake of PAMAM-PEG-SRL/DNA (Figure 3) was more than that of PAMAM/DNA. Our data also showed that the use of inhibitors such as phenylarsin oxide, filipin, or lactoferrin (Figure 3) resulted in the inhibition of cellular uptake in the PAMAM-PEG-SRL/DNA complex. These inhibitors were used to study the mechanism blocking the entry of nanoparticles into the cells. Among them, lactoferrin and phenylarsin oxide have the highest level of inhibition (Figure 3). Lactoferrin, as a ligand, is known to have a high affinity to LRP (20, 32). As depicted in Figure 3, Lactoferrin, as an LRP inhibitor, is able to inhibit gene delivery, and shows the increased inhibition of nanoparticle entry to cells (Figure 3E, F). 

**Figure 4:**
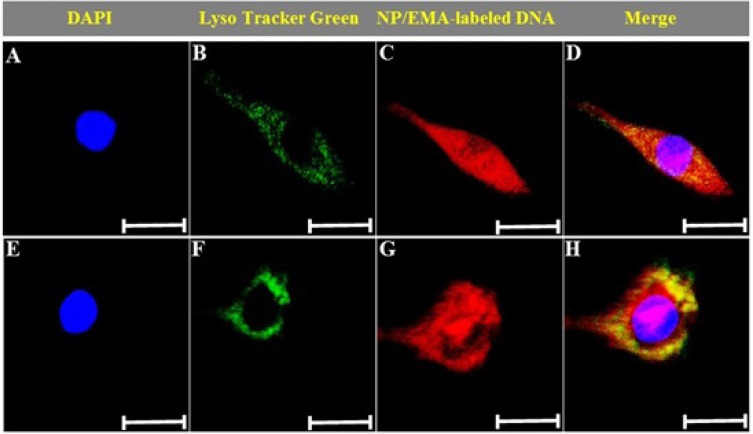
Cellular loclization of PAMAM–PEG–SRL/DNA nanoparticles. Photos were taken after cells incubated with nanoparticles for 15 min (A–D), or 60 min (E–H). Green: Lysotracker Green (B, F). Red: EMA-labeled DNA (C, G). Yellow: Lysotracker Green colocalized with EMA-labeled DNA (D, H). Bar: 10μm

**Figure 5:**
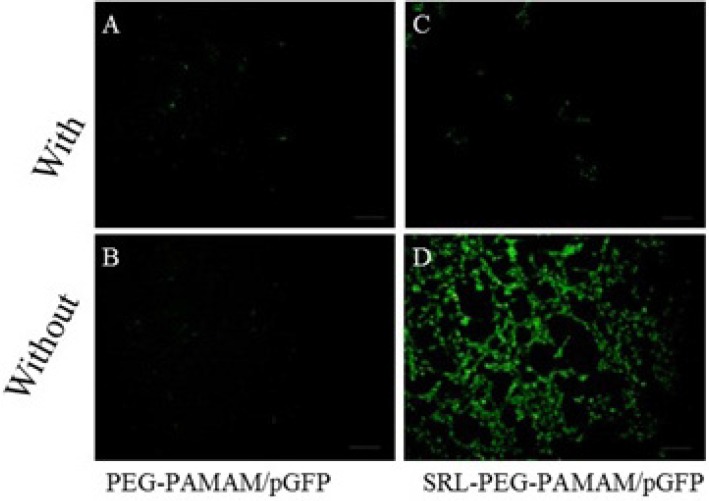
Gene expression on C6 glioma cells. The fluorescence images of GFP expression were taken 48 h post-infection with PAMAM-PEG/DNA and PAMAM-PEG-SRL/DNA with or without lactoferrin. Bar: 50μm. Results were performed as fluorescent microscopy images

**Figure 6:**
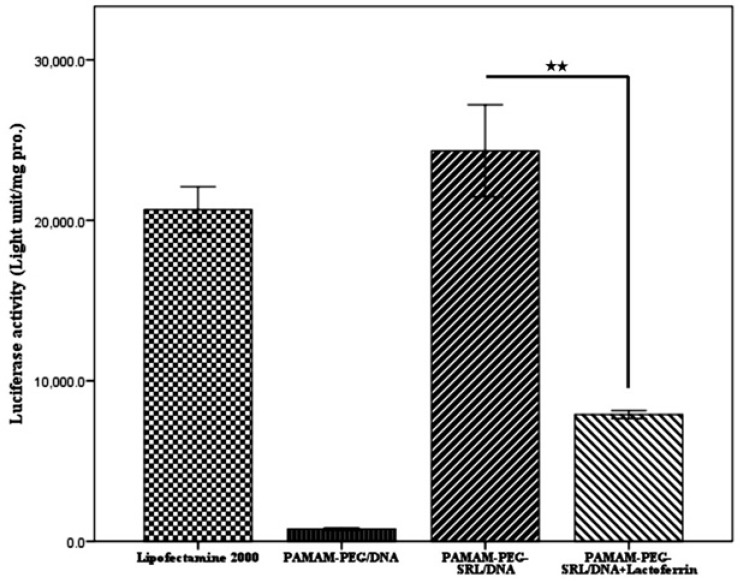
The quantitative evaluation of gene expression in vitro. Luciferase activity was measured 48 h post-transfection and expressed as light units per mg pro. **p < 0.01, compared with the PAMAM-PEG/DNA and PAMAM-PEG-SRL/DNA+Lactoferrin nanoparticles. Data represent the mean ±S.E.M (n=6

*Cellular localization of PAMAM–PEG–SRL/DNA nanoparticles*

The confocal microscopy was used to study the intracellular distribution of PAMAM-PEG-SRL/DNA nanoparticles. By definition, endocytosis is a process where molecules are internalized as vesicles into the cytoplasm, so-called endosomes (33). As a general rule, drugs should escape endosome to be able to act in the cell; otherwise, they will end in lysosomes, where they will be destroyed. Therefore, escape in early stages (endosomal escape) is necessary for drugs entered into the cells via endocytosis. The cellular uptake of EMA-labeled PAMAM-PEG-SRL/DNA complexes occurred in a time-dependent manner. As shown in Figure 4. the small red dots represent DNA, whereas the small green dots indicate LysoTracker green. Because LysoTracker green marks the endosomes or lysosomes, the green and red spots are co-localized inside endosomes and lysosomes after 15 min (Figure 4E-H), indicating that DNA has entered into the cell through endocytosis (as endolysosome cargo in endosomes and lysosomes). After 60 min, however, the red dots, the larger part of DNA illustrated in Figure 4I-L, were dispersed throughout the whole cell, as can be found while entering the nucleus). This shows that nanoparticles have escaped endosomes and lysosomes (32). This phenomenon has been described as proton sponge theory (34, 35). Based on this theory, secondary and tertiary amines of cationic carriers resist endosomal acidification by capturing and absorpting protons. This function leads to a delay in lysosomal fusion to the endosomes, and enables counterions (e.g. Cl−and H2O) to flood the endosomes. Entrance of water into the endosomes result in the swelling and rupture of endosomes, and the release of cargo into the cytoplasm (36). Although the mechanism underlying the pDNA entrance to the nucleus is not yet clearly understood, it seems that pDNA released from late endolysosomes enters into the nucleus by the diffusion process (29).

*Qualitative analysis of In-Vitro transfection efficiency in C6 glioma cells*

To qualitatively analyze in vitro gene expression efficiency of the synthesized nanoparticles, the C6 glioma cells were exposed to PAMAM-PEG/pDNA and PAMAM-PEG-SRL/pDNA with or without lactoferrin (Figure 5). Our data showed that, without lactoferrin, PAMAM-PEG-SRL/pDNA induces higher GFP formation than PAMAM-PEG/pDNA (Figure 5C, D). Meanwhile, the presence of lactoferrin decreased the GFP expression in C6 glioma cells treated with PAMAM-PEG-SRL/pDNA (Figure 5B). This phenomenon occurred possibly due to the higher affinity of lactoferrin to LRPs than the SRL peptide. Finally, lactoferrin was found to have no effect on PAMAM-PEG/pDNA.

*Quantification of gene transfection efficiency in C6 glioma cells*

Gene transfer efficiency in cancer cells was investigated by using luciferase gene expression, and the results were reported as the lighting Lux unit per mg of protein (RLU/mg). As illustrated in Figure 6 A and B. the optical expression of the PAMAM-PEG-SRL nanoparticles at a polymer/DNA ratio of 10:1, and Lipofectamine reached 2.38×10^5^ and 2.1×10^5^ RLU/mg of the protein, respectively. However, in the presence of lactoferrin, the expression level decreased to 8×10^4^ RLU/mg protein, which might be due to the competitive inhibition effect of lactoferrin on the uptake of SRL modified nanoparticles.

## Discussion

Glioma, one of the most common primary brain tumors, is categorized into four groups according to WHO classifications (37). Although its prevalence remains lower than other solid tumors, glioma has a very weak prognosis (38). Development of an effective therapeutic delivery system is crucial to selectively deliver cytotoxic drugs to the tumor cells while sparing normal cells. A dual-targeting delivery system, currently thought to be a promising approach for gene delivery to brain tumors, confers a series of advantages, such as high efficiency, low toxicity, stability and high transfection efficiency. These advantages may provide new opportunities in enhanced delivery of therapeutic agents across the blood–brain barrier to treat brain diseases (11, 15). A variety of ligand types has been used for dual-targeting, such as lactoferrin (11), Angiopep (9), Chlorotoxin (12), RGD (13), LDLR-mediated peptide-22 (14), and T7 peptide (15). More recently, we have synthesized the SRL-modified PAMAM nanocarrier for gene delivery to the brain (24). In addition, we have demonstrated that the SRL peptide serves as a LRP ligand. Because C6 glioma cells are able to overexpress LRP (10), we sought to use our newly-synthesized nanocarrier as a new dual targeting system for gene delivery to the glioma cells. In the present study, PAMAM G4 serves as a primary gene delivery vehicle, while SRL through residues added to the end of N binds to the dendrimer using NHS-PEG-MAL. 

The cellular absorption of SRL-modified nanoparticles is dependent upon dose and energy contents (Figure 1 and 3). 

Nowadays, the non-invasive gene delivery to the brain remains a challenge. However, receptor mediated transcytosis (RMT) is a potential way to successfully transfer drugs and genes across the BBB using endogenous or synthetics ligands (39, 40). Over the past few years, numerous attempts have been made to produce the targeted LRP peptides (angiopep-2) (27, 41) being able to identify other types of this ligand (LDLR) (42, 43). The physiological role of LRP receptors is to control BBB permeability, vascular tone and the postischemic lesion formation as a response to activated tissue-type plasminogen activator (18). LRP is expressed on all cells, and associated with more than 40 different types of ligands such as lipoproteins, protease/protease-inhibitor complexes, extracellular matrix proteins, viruses, growth factors and cytokines (19). A fast transcytosis rate is the unique feature of this protein (21). Lipoprotein endocytosis is mediated by three structurally related cell surface receptors, which the presence of at least one copy of an NPXY motif in their structure leads to the receptor internalization into coated pits (16). LRP is one member of the LDL receptor gene family. In our recent experiment, we showed that SRL peptide has a competitive inhibitory effect on the cellular uptake of PAMAM-PEG-SRL nanoparticles, consistent with the findings of Pasqualini and our recent work (24, 25). 

As we shown in our recent paper, the entry of PAMAM-PEG-SRL/DNA complexes can be affected by different mechanisms (24). It is likely that uptake mechanisms can be affected by complex formation. Therefore, to study the entry route of PAMAM-PEG-SRL/DNA nanoparticles to C6 glioma cells, we attempted to use endocytosis inhibitors such as, filipin complex (endocytosis inhibitor mediated through caveolae) (44), colchicine (macropinocytosis inhibitor) (45) and phenylarsine oxide (endocytosis inhibitor mediated through clathrin). The findings showed that all the inhibitors were able to prevent the entry of nanoparticles into the cells the same BCECs as in our previous work, indicating involvement of both routes with the mediation of the receptor (RMT) in the cellular uptake of these nanoparticles. Among the inhibitors, the highest level of inhibition was found through the phenylarsine oxide pathway (Figure 3), observed in nanoparticles modified by other LRP ligands, such as lactoferrin and angiopep-2 (46-48). After comparing the sequences of the identified LDL ligands, such as angiopep-2 (TFFYGGSRGKRNNFKTEEY) (27), LDLR-mediated peptide-22 ( MPRLRGC) (42), and the SRL peptide (CLSSRLDA) (owing the fact that both linear forms of the peptide had the same cellular uptake characteristics, as mentioned earlier), we suggest that these three peptides indicated a relatively similar section (underlined letters), and this amino acid sequence seems to play an important role in identifying LDL receptors. However, more investigations are required in this area to clarify the exact mechanism. Furthermore, our qualitative and quantitative gene transfection analysis showed that SRLs has a very low affinity to LRPs, as compared to lactoferrin. On the other hand, SRL-modified nanoparticles, compared to lipofectamine, exhibited high efficiency as a standard carrier, while not significant.

Finally, as LRP receptors are expressed in some other types of cancers such as colorectal carcinoma, the current nanoparticles may be used for increased delivery and targeting of anticancer drugs to these sites. It is obvious that for such kind of delivery, special consideration and enhancement techniques are required (49).

## Conclusion

A new kind of modified G4 PAMAM conjugating on the surface of the SRL peptide was prepared and demonstrated to be efficient for glioma targeting gene delivery in vitro. In vitro qualitative and quantitative experiments by using the confocal microscopy, fluorescent microscopy, flow cytometry and AFM showed that PAMAM-PEG-SRL is able of targeting the C6 glioma cells with higher capability than BCECs. PAMAM–PEG–SRL was internalized by C6 glioma cells through clathrin and caveolae-mediated endocytosis, similar to BCECs. LRP-mediated endocytosis may be the main cellular mechanism for SRL-modified nanoparticles internalization. We believe that PAMAM–PEG–SRL has a promising potential for an efficientdual targeting gene delivery system to target the brain and glioma.
